# Assigning and visualizing germline genes in antibody repertoires

**DOI:** 10.1098/rstb.2014.0240

**Published:** 2015-09-05

**Authors:** Simon D. W. Frost, Ben Murrell, A. S. Md. Mukarram Hossain, Gregg J. Silverman, Sergei L. Kosakovsky Pond

**Affiliations:** 1 Department of Veterinary Medicine, University of Cambridge, Madingley Road, Cambridge, Cambridgeshire CB3 0ES, UK; 2 Department of Medicine, University of California, San Diego, La Jolla, CA 92093, USA; 3 Department of Medicine, New York University School of Medicine, New York, NY 10016, USA; 4 Department of Pathology, New York University School of Medicine, New York, NY 10016, USA

**Keywords:** immunoglobulin, V(D)J rearrangements, phylogenetic models, classification, visualization, IgSCUEAL

## Abstract

Identifying the germline genes involved in immunoglobulin rearrangements is an essential first step in the analysis of antibody repertoires. Based on our prior work in analysing diverse recombinant viruses, we present IgSCUEAL (Immunoglobulin Subtype Classification Using Evolutionary ALgorithms), a phylogenetic approach to assign V and J regions of immunoglobulin sequences to their corresponding germline alleles, with D regions assigned using a simple pairwise alignment algorithm. We also develop an interactive web application for viewing the results, allowing the user to explore the frequency distribution of sequence assignments and CDR3 region length statistics, which is useful for summarizing repertoires, as well as a detailed viewer of rearrangements and region alignments for individual query sequences. We demonstrate the accuracy and utility of our method compared with sequence similarity-based approaches and other non-phylogenetic model-based approaches, using both simulated data and a set of evaluation datasets of human immunoglobulin heavy chain sequences. IgSCUEAL demonstrates the highest accuracy of V and J assignment amongst existing approaches, even when the reassorted sequence is highly mutated, and can successfully cluster sequences on the basis of shared V/J germline alleles.

## 1. Introduction

Vertebrates have evolved sophisticated mechanisms of immunity in response to pathogens, which as a consequence of their typically shorter generation time, place significant selection pressure on their hosts to respond on a commensurate time scale. Antibodies, which can block infection through binding [1], are generated through rearrangement of germline genes, with subsequent somatic mutations that result in a potentially diverse repertoire of antibodies that can combat pathogens that themselves may exist as a diverse swarm, or ‘quasi-species’. Indeed, the immune system is capable of producing such a diversity of somatically generated antibody gene sequences that it can exceed by many orders of magnitude the total number of lymphocytes present in the host.

With the advent of high-throughput sequencing platforms, insights can be gained into the microevolutionary events that shape antibody repertoires, and into the underlying mechanisms [2]. This information can be used to aid vaccinology studies through a mechanistic understanding of how exposure to an antigen may lead to immunity, and can yield insights into the pathogenesis of disorders such as acute lymphoblastic leukaemia, chronic lymphocytic leukaemia and systemic lupus erythematosus.

Diversity in the immunoglobulin heavy chain (IGH) repertoire is generated by four processes: combinatorial choice of V, D and J regions; deletions in the V, D and J regions; addition of palindromic (‘P’-) and non-templated (‘N’-) nucleotides at the junctions; and somatic hypermutation [3]. As a result, repertoires are composed of clonotypes with different germline origins. Characterization of these clonotypes allows us to assess how much diversity in the repertoire is due to germline variation within V, D and J genes at the population level, as well as to determine the extent of somatic hypermutation. The importance of dividing a repertoire into clonotypic ‘building blocks’ depends on the application. In B-cell lymphoma, assessment of the mutational status of V regions is relevant in determining whether tumour cells originate from virgin B cells or from germinal centre and postfollicular B cells. Identification of biases in gene usage is also relevant in the study of autoimmune diseases. Some microbial pathogens produce super-antigens which target relatively conserved motifs in a large swath of the repertoire, tracing their origins to a subset of the V genes; in these applications the assignment of individual IGH sequences to V(D)J rearrangements is of primary interest [4].

**Figure 1.**
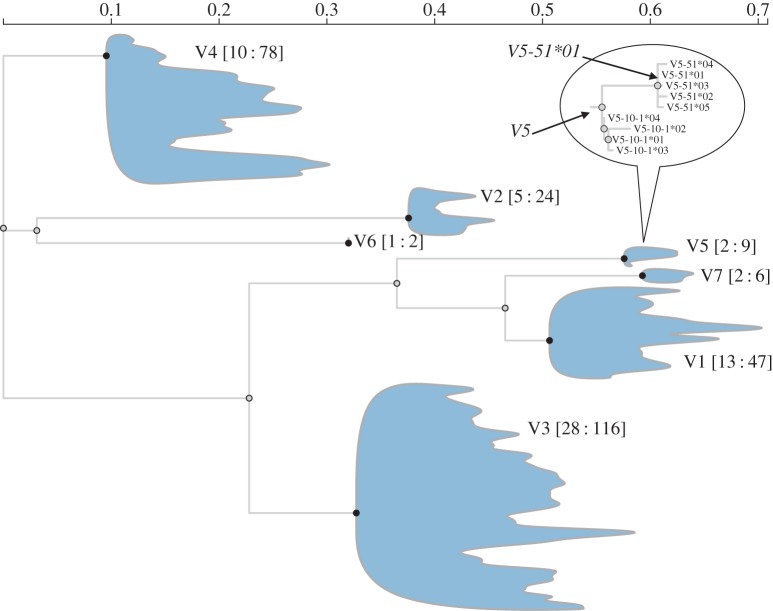
A maximum-likelihood phylogeny of unique functional (F and ORF) germline V genes. Individual family clades have been collapsed to represent the tree more compactly, while showing the diversity encompassed by the clade. The counts of unique family members (*01 alleles) and total allelic variants are shown as the first and second numbers following family names, respectively. The clade for the V5 family is shown in the enlarged inset, and demonstrates some conventions used for assigning labels to internal nodes in the tree. For example, the internal node V5-51*01 inherits its label from a child node with a branch length of zero. In the context of phylogenetic likelihood, this implies that the sequence at the internal node is identical to that of the descendant node, justifying label propagation. The parent of the V5-51*01 internal node is labelled *V5*, because it is the most fully resolved label shared by all of its descendants (V5-51*xx and V5-10*xx alleles), and none of its children have branch lengths of zero. The main body of this figure, as well as of figures 2 and 4, were generated using an interactive web application used to view IgSCUEAL results.

To start to fully define a clonotype, regions of immunoglobulins that originate from V, D and J genes must be identified and assigned to their respective germline genes. Methods for V(D)J assignment fall into two classes: alignment-based methods (JOINSOLVER [5], IMGT/V-Quest [6,7], Ab-origin [8] and IgBLAST [9]), and model-based methods (e.g. iHMMuneAlign [10], SoDA [11], and SoDA2 [12] which are based on Hidden Markov Models, and others [13,14]). However, none of these approaches take the phylogenetic relationship between germline genes into account, which is particularly prominent for V genes (figure 1); in fact, V gene families form distinct phylogenetic clades which recapitulate the original delineation based on amino acid sequence similarity. A phylogenetic approach to V(D)J assignment may be useful in a number of ways: firstly, as probabilistic models of evolution can be used, it is possible to quantify the uncertainty with which a query sequence is assigned to a particular germline; secondly, this approach permits a query sequence to cluster with an ancestral sequence. This may occur when sequences are highly mutated, such that the identity of the germline alleles is obscured by saturation of mutations, or when the reference set of germline sequences is incomplete. While the human and mouse genomes have been mapped extensively, there is increasing interest in analysing immunoglobulin repertoires for other species for which the genomes have not been fully annotated. For example, while only 23 IGHV annotated genes exist in the IMGT database for the rhesus macaque *Macaca mulatta* [15], 63 IGHV-like sequences have been identified in the macaque genome using a bioinformatics approach [16].

Application of these tools to data from mass sequencing platforms yields a glut of information that is difficult to digest. Binning of millions of reads into unique V(D)J rearrangements is important both as a sensible approach to data reduction (clustering similar reads), and as a means to pull out a subset of the repertoire that is of specific interest, e.g. all those sequences that match a pre-defined rearrangement, for instance as is now common in HIV-1 vaccine research [17]. Interactive tools that allow the user to explore the composition of immunoglobulin repertoires can help to interpret repertoire sequencing (‘Rep-Seq’) data in a more manageable way. Even how an assignment is reached for an individual sequence may be of interest, especially for heavily mutated sequences that have diverged substantially from the germline.

Our aims are twofold: firstly, we present a phylogenetic approach to identifying recombination breakpoints and assigning germline genes from rearranged immunoglobulin genes. By using a model of substitution, we can generate a quantitative comparison of different V(D)J assignments, while the use of a phylogeny allows for the possibility that the true germline alleles are absent from the reference data. Secondly, we demonstrate interactive visualizations of rearrangements in antibody repertoire data, as well as a detailed viewer of rearrangements for an individual sequence. We apply our approach to simulated data, to data from genotyped individuals and to clonal data.

## 2. Material and methods

### (a) Obtaining reference sequence data

Sequences of human IGHV, IGHD and IGHJ were downloaded from IMGT (http://www.imgt.org/vquest/refseqh.html), using reference directory release 201443-5 (24 October 2014), and periods in these datasets, introduced in order to achieve a consistent numbering scheme for immunoglobulins, were removed. Protein displays of IGHV were also downloaded (http://www.imgt.org/IMGTrepertoire/Proteins/index.php), which gives the boundaries for the framework and complementarity determining regions (FR1–3, CDR1–3) for each of the primary (*01) alleles. We restricted our analysis to functional genes and open reading frames (ORFs), resulting in 290 V genes, 44 D genes and 13 J genes.

### (b) Generating a reference alignment for IgSCUEAL

As IgSCUEAL uses a phylogenetic approach to assign V and J regions, the algorithm requires a multiple sequence alignment (MSA); specifically, we employ a codon-based MSA, which allows us to employ more biologically realistic codon-based substitution models when reconstructing ancestral sequences, subsequently used by IgSCUEAL for query homology matching and alignment (see §2c,d). V genes were aligned using a codon-based algorithm implemented in MACSE v. 1.01b [18], and J genes were aligned in nucleotide space using MUSCLE v. 3.8.31 [19], with further manual refinements; codon alignment was found to be necessary for V gene sequences, despite the increased computational expense and manual alignment tuning, which we found necessary when using MACSE. Duplicate sequences (after excluding gaps) were filtered from the alignment, resulting in a reduction in the V genes for the human reference dataset to 282 functional genes plus ORFs. Phylogenetic trees were reconstructed separately for the V and J alignments using CodonPhyML [20], and rooted in a way that separates individual families (e.g. V1, V2, etc.) into complete clades that are descendant from the root, and does not make single sequences direct descendants of the root. V and J alignments were merged into a ‘block-matrix’ format. The merged alignment was augmented with computationally derived most recent common ancestors (MRCAs) for V and J alleles. Each terminal branch in the trees for V and J regions was annotated with the corresponding germline allele (e.g. V5-51*01), and each internal branch was assigned a parsimony-derived classification based on the labelling of its descendant branches (figure 1). D allele sequences were included separately as a dictionary in a HyPhy batch language file, for matching via an alignment approach. We considered both forward and inverted D sequences, although the latter may play only a minor role in shaping IGH diversity [13].

### (c) Mapping sequence regions

The query immunoglobulin sequence is codon-aligned to the set of user-designated references, which always includes the MRCA of V and J segments (i.e. no D) reconstructed using joint maximum likelihood under the MG94 × GTR model of evolution fitted to the topology generated by CodonPhyML; this sequence is inferred once during reference construction, and is the same for all queries. For the F + ORF reference set used here, we further aligned the query to all combinations of *01 alleles of IGHV and IGHJ, and selected the mapping yielding the best homology score. Because the reference alignment encodes FR and CDR, we segment the query sequence into corresponding regions based on how it maps to the reference alignment. A productive rearrangement (i.e. one without premature stop codons) is inferred if an in-frame junction region can be extracted. The junction region is defined as spanning the sequence from the 3′ cysteine in the germline FW3 region, to either the beginning of the J-region as defined by the ‘[FW]G.G’ regular-expression motif in the J region, or—should the motif be absent—to the position preceding the conserved tryptophan in the MSA of the J region.

### (d) Rearrangement classification

**Figure 2.**
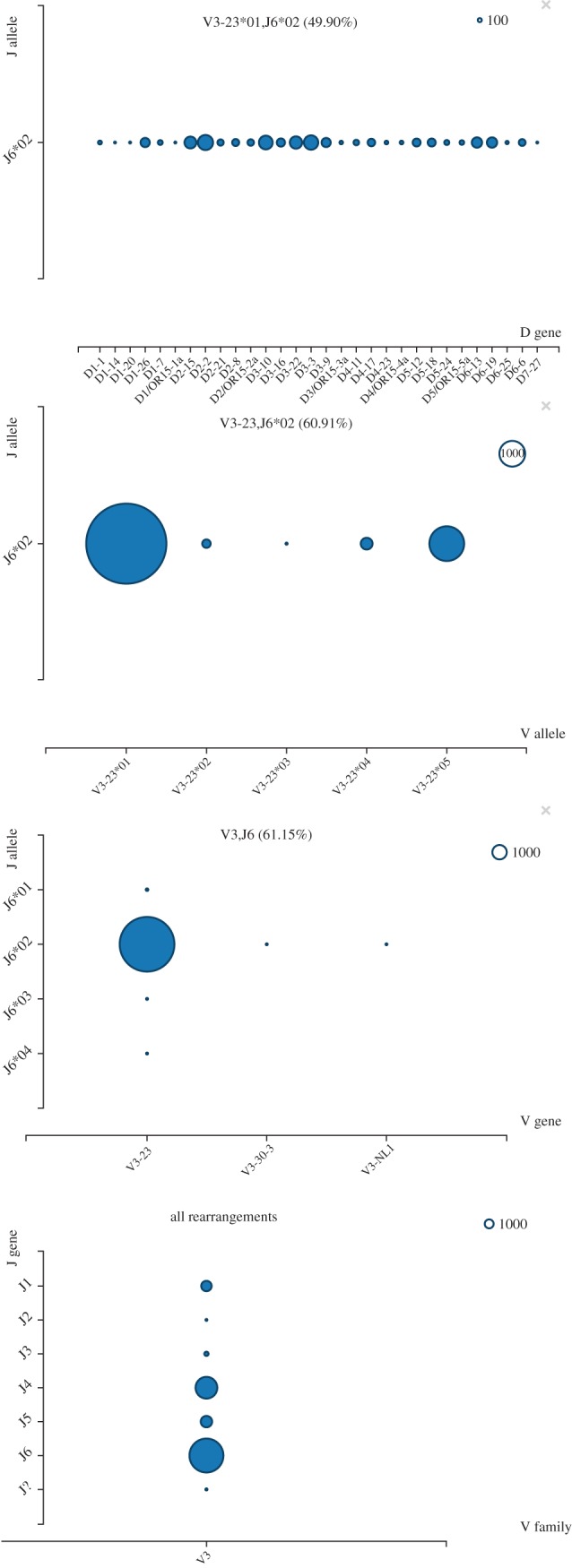
A visualization of the rearrangements inferred by IgSCUEAL for the 6329 immunoglobulin sequences from Ohm-Laursen *et al.* [13]. The four plots show the proportions of sequences inferred for (bottom to top): all rearrangements, rearrangements assigned to the IGHV3 family and IGHJ6 gene (61.15% of total), rearrangements assigned to the IGHV3-23 gene and IGHJ6*02 allele (60.19%) and those assigned to the IGHV3-23*01 and IGHJ6*02 alleles (49.90%).

Our algorithm is an adaptation of the previously published SCUEAL (Subtype Classification Using Evolutionary ALgorithms) method [21], originally developed to classify HIV-1 isolates into pure and mosaic (recombinant) subtypes. An early implementation of IgSCUEAL has been used to classify V*κ* L chain transcripts in mice exposed to a B-cell superantigen [22]; we have refined the approach significantly, and in this study, we focus on reassortments involving the human IGH locus, although the approach is more widely applicable to a range of species and loci. The algorithm proceeds in the following stages (see figure 2 for an example). Firstly, the CHC genetic algorithm [23] based on elitist selection, rapid mixing through free recombination, and small population size (needed for computational tractability), is used to search for the best fitting model that encodes the placement of the query V segment in the V reference tree, the placement of the query J segment in the J reference tree, and the location of the breakpoint separating the sequence into the V and J segments. The fitness of each model is determined by the small-sample AIC (AIC_c_) score based upon the fit of the GTR nucleotide substitution model to the sequence alignment. Although in principle, we could use the MG94 × GTR codon substitution model used to fit the reference alignment and generate ancestral sequences, this would slow down IgSCUEAL by two orders of magnitude, and is unlikely to give dramatically different results. Owing to the time reversibility of the model, the problem of optimizing the phylogenetic likelihood when one sequence is attached to a fixed reference tree at a given branch, is computationally equivalent to optimizing branch lengths in three-taxon trees, and can, therefore, be run largely independently of the size of the reference tree. Secondly, upon convergence, each model considered by the CHC algorithm is assigned an Akaike weight: exp[−0.5(AIC_c_ − min AIC_c_)], scaled so that all model weights add to unity, which is then interpreted as evidence in support of this model. The V–J combination with the highest total model weight is reported as the inferred rearrangement, and all other rearrangements with at least 0.01 Akaike weight are reported as alternatives. Thirdly, the D allele which has the highest nucleotide alignment score (computed using the standard Smith–Waterman algorithm with affine gap penalties) with the junction region of the query is reported as the inferred allele; in the case where multiple D alleles yield the same alignment score, they are all reported as equally likely.

### (e) Clustering rearrangements

Inferred rearrangements are often used to help operationally to define a clonotype [24,25]. For putatively clonal sequences, we constructed a network where nodes represent each sequence and edges between nodes represent whether there are rearrangements in common between each sequence's credible set. The distribution of strongly connected components of this network was used to summarize the extent to which assignments cluster together clonally derived sequences.

### (f) Comparison with other software

We compared IgSCUEAL with several other tools, including IMGT/HighV-QUEST v. 1.3.1 [26], IgBLAST v. 1.4.0 [9] and iHMMune-Align (1-06-2007) [10]. A binary version of SoDA (Somatic Diversification Analysis, v. 1.1) was extracted from Automation [27], and Python scripts were used to run SoDA for each query sequence and to parse the output. We also used vdjalign (http://github.com/cmccoy/ighutil), a simple Smith–Waterman alignment-based program previously used in an evolutionary analysis of immunoglobulin repertoires [28], vdj (http://github.com/laserson/vdj), as used in [25] and Cloanalyst (v. 2015/1), a model-based approach using algorithms described in [14]. With the exception of vdj and vdjalign, which used a larger reference dataset, all programs employed the same F + ORF reference dataset. Uncertainty in IgBLAST was accommodated by considering the top three hits, while the IMGT/HighV-QUEST reports a list of assignments of variable length.

### (g) Datasets

#### (i) Simulated data

We developed a simulation program to generate rearranged immunoglobulin sequences from the human germline IGHV (*n* = 282), IGHD (*n* = 44) and IGHJ (*n* = 13) sequences, considering only unique alleles present in the IgSCUEAL reference alignment. We first considered simple rearrangements in which all possible combinations of IGHV, IGHD and IGHJ *01 alleles were considered, obtained by concatenating the germline sequences (*n* = 12 060). We also simulated a set of 10 000 sequences under a more realistic model that included random deletions and addition of N-nucleotides. Alleles were sampled at random, using the number of alleles per gene as inverse weights, in order to avoid oversampling genes with many alleles. The lengths of deletions in the 3′ end of the V region, the 5′ end of the J region, and both ends of the D region as well as the lengths of the N1 and N2 regions were drawn independently from the distributions inferred by Jackson *et al.* [29]. The base composition of the simulated N1 and N2 regions was sampled from an empirical distribution obtained from a concatenation of the inferred N1 and N2 regions. Simulated regions were retained if the rearranged gene was (i) free of stop codons, (ii) contained a CDR3 region recognizable by the regular expression proposed by D'Angelo *et al.* [30] and (iii) had an in-frame J region, with the conserved ‘[FW]G.G’ and ‘TVSS’ motifs. Mutations were also added using the S5F model proposed by Yaari *et al.* [31]. Simulated unmutated sequences were split into 5-mers, and mutability for each 5-mer calculated; the mutability of the first two and last two positions was set to zero. The position of the mutation was randomly chosen using mutability values as weights. Given a mutation at a position, the base was randomly chosen based on the probabilities, given the 5-mer, according to the S5F model. Two datasets were generated with either 40 or 80 mutations per sequence, using the 10 000 unmutated sequence dataset as ‘seed’ sequences. We also generated 100 clonal datasets by randomly selecting 100 unmutated rearranged sequences (including insertions and deletions), ‘cloning’ each sequence 100 times and adding either 40 or 80 mutations per sequence.

#### (ii) Datasets from genotyped individuals

The Stanford S22 dataset has been used previously to benchmark human antibody gene alignment utilities [32]. This dataset was derived from an individual who had been fully genotyped; the performance of a utility is determined by the proportion of sequences that are assigned a germline gene that is absent from the individual. A FASTA-formatted dataset containing 13 153 sequences was obtained from http://www.emi.unsw.edu.au/~ihmmune/IGHUtilityEval ; this website was also used to download results from other software packages (e.g. SoDA2) that were not available at the time of writing, and to evaluate assignments from IgSCUEAL. Following [13] and [8], we analysed a set of 6329 clonally unrelated IGHV3-23-IGHD-IGHJ rearrangements, obtained from individuals homozygous for IGHV3-23*01 and IGHJ6*02, and amplified using primers intended to be specific for IGHV3-23; the original study also amplified a number of IGHV3-h pseudogenes, which were excluded from the analysis.

#### (iii) Clonally related datasets

Clonally related datasets provide a means to assess both germline accuracy and identification of junctions. While the exact V(D)J regions are unknown, all sequences should share the same assignment, as well as junction locations. We used two datasets derived from IgD+ IgM-CD38+ B cells, one of 57 sequences (GenBank accessions AF262145–AF262201, extracted from PopSet 8810007 [33]) and one of 106 sequences (GenBank accessions EF544883–EF544988 [34]). We also analysed 11 sequences from an HIV-infected individual, donor N152, the source of the broadly neutralizing antibody 10E8 [35], which represents a highly mutated (*ca* 20% divergence from germline) clonal dataset [36] (GenBank accessions KC754704–KC754714). In order to reduce the effect of somatic hypermutations, we also generated ancestral reconstructions of the sequences using a centre-of-tree (COT) approach, as implemented in the DIVEIN server [37]; COT sequences were derived from MSAs of the clonal datasets obtained using MACSE v. 1.01b [18]. Given a nucleotide alignment, DIVEIN uses PhyML (v. 3.0.1 [38]) to obtain a maximum-likelihood tree, assuming a GTR model with rate heterogeneity modelled as a discretized gamma distribution with four categories plus a proportion of invariant sites.

### (h) Implementation

IgSCUEAL is implemented as a HyPhy [39] batch file, with additional processing via Python 3 scripts, included in the IgSCUEAL distribution. Sequences can be screened in parallel using a message passing interface (MPI) implementation, achieving throughput amenable to NGS datasets. We have developed several standalone web applications so that IgSCUEAL output can be more readily explored and interpreted. Our applications run in any modern web browser and consume JSON files generated by various modules of IgSCUEAL. They are based on popular open-source libraries, including jQuery, d3 and Bootstrap, and are implemented in HTML5 and JavaScript. Figures 1, 2 and 4 supply examples of outputs generated by these tools. The simulation programs were written in Python. The versions used in this manuscript, along with the evaluation datasets, are included in the electronic supplementary material, and an up-to-date version of the software and public server instance hosting web visualization applications are linked from http://antibodyo.me.

## 3. Results

The reliable assignment of rearranged immunoglobulin genes to their respective germline alleles is an important first step in the analysis of antibody repertoires, to determine germline gene usage, as well as to help characterize clonotypes within the repertoire. Like other methods, our approach, IgSCUEAL, employs reference datasets of germline alleles, but unlike other approaches, we assume that immunoglobulin gene families for a given region are related to one another. In addition, we employ a statistical model of sequence evolution, which allows us to generate a weighted set of assignments, rather than a single ‘best hit’, which we use as the basis of a simple algorithm to identify sequences that may be clonally related (or strictly speaking, to separate sets of sequences that are not clonally related). In most biological datasets, the true germline alleles are not known, so in order to evaluate our method, we make use of both simulated data and some more unusual biological datasets that can provide insights into the performance of V(D)J assignment algorithms.

### (a) Simulated data

We first explored the accuracy of our method using a suite of simulated datasets where the correct rearrangements are known. In addition to rearranged sequences, IgSCUEAL can also classify V genes. IgSCUEAL correctly identified all V alleles (allowing for genetically identical alleles to be mapped to their single representative in the reference alignment), although the support, in terms of Akaike weights, varied by allele. This reflects differences in the ability to discriminate between alleles that arise because of the phylogenetic structure of the V gene reference alignment.

For V(D)J rearrangements, we considered three scenarios: firstly, simple rearrangements were generated by concatenating V, D and J germline genes (*01 alleles only), resulting in 12 060 possible combinations. Similarity-based methods such as IgBLAST [9] are expected to work well in this scenario, as an exact match to the germline is possible if the alignment to the regions is correct. IgSCUEAL exhibited similar accuracy to IgBLAST in this setting for V and J regions (the few misclassifications for V and J are because of the stochastic nature of the GA where it failed to converge to the correct solution).

Secondly, we considered rearrangements with insertions and deletions: we deleted the 3′ region of the V gene, the 5′ region of the J gene, and both the 5′ and 3′ ends of the D gene, and N-nucleotides were added between the V and D regions, and between the D and J regions. As described in Material and methods §2g(i), the lengths of the insertions and deletions, as well as the base composition of the N-nucleotides, were taken from biological data [29]. Ten thousand simulated sequences were generated using all functional and ORF germline sequences. IgSCUEAL makes only four mistakes in classifying the V allele, and always misclassifies the gene only once (table 1). In a small proportion of cases (1.46%), the correct allele is not the one given the strongest phylogenetic support, but the correct one is included in the list of alternative assignments. The J allele is classified correctly in 99.7% of cases. Indel variation dilutes the already weak signal for the D region (especially for the shorter alleles), causing IgSCUEAL to misclassify the D allele in 29% of cases. For V and J alleles, however, the performance of IgSCUEAL is similar to IgBLAST.

**Table 1.** Comparative method performance on simulated data. The *correct* column reports the proportion of sequences where the correct germline allele received the highest model-averaged support (IgSCUEAL) or was the single result reported by other tools, or where correct allele received greater than 1% model-averaged support in IgSCUEAL, but a different allele was inferred to have the highest support (for other methods, this is taken to include the cases when multiple assigned alleles included the correct one). The latter proportion is indicated in parentheses. The proportion of cases for which one of the three regions was not assigned a germline are tabulated in the *no assignment* column. The *wrong* column reports the proportion of miscalled sequences (either allele, gene), with the proportion of sequences assigned to the wrong gene indicated in parentheses.

	correct (alternative), %			wrong (gene), %			no assignment, %		
method	V	D	J	V	D	J	V	D	J
simple rearrangements									
IgSCUEAL	99.93 (7.60)	97.12 (0.00)	99.99 (0.32)	0.07 (0.07)	2.88 (2.78)	0.01 (0.00)	0.00	0.00	0.00
IgBLAST	100.00 (3.68)	100.00 (23.33)	100.00 (0.00)	0.00 (0.00)	0.00 (0.00)	0.00 (0.00)	0.00	0.00	0.00
iHMMune	100.00 (0.00)	100.00 (0.00)	100.00 (0.00)	0.00 (0.00)	0.00 (0.00)	0.00 (0.00)	0.00	0.00	0.00
SoDA	98.96 (0.00)	100.00 (0.00)	100.00 (0.00)	1.04 (0.00)	0.00 (0.00)	0.00 (0.00)	0.00	0.00	0.00
V-Quest^a^	100.00 (10.91)	100.00 (0.00)	100.00 (0.00)	0.00 (0.00)	0.00 (0.00)	0.00 (0.00)	0.00	0.00	0.00
Clonanalyst^a^	99.92 (0.00)	100.00 (0.00)	100.00 (0.00)	0.08 (0.05)	0.00 (0.00)	0.00 (0.00)	0.00	0.00	0.00
vdj^a^	95.89 (0.00)	100.00 (0.00)	100.00 (0.00)	4.11 (0.00)	0.00 (0.00)	0.00 (0.00)	0.00	0.00	0.00
vdjalign^a^	99.90 (0.00)	100.00 (0.00)	100.00 (0.00)	0.10 (0.00)	0.00 (0.00)	0.00 (0.00)	0.00	0.00	0.00
rearrangements with insertions and deletions									
IgSCUEAL	99.94 (4.95)	70.01 (3.96)	99.69 (3.41)	0.06 (0.01)	29.99 (28.51)	0.31 (0.28)	0.00	0.00	0.00
IgBLAST	99.66 (2.85)	83.23 (27.98)	99.85 (0.86)	0.34 (0.01)	16.77 (16.50)	0.15 (0.11)	0.00	0.00	0.00
iHMMune	98.86 (0.00)	74.51 (0.00)	98.28 (0.00)	0.40 (0.00)	11.41 (9.07)	0.98 (0.74)	0.74	14.08	0.74
SoDA	98.57 (0.00)	77.56 (0.00)	94.52 (0.00)	1.43 (0.54)	22.44 (20.07)	5.48 (1.26)	0.00	0.00	0.00
V-Quest^a^	100.00 (5.16)	74.17 (0.00)	97.85 (2.61)	0.00 (0.00)	23.71 (20.80)	2.12 (2.00)	0.00	2.12	0.03
Clonanalyst^a^	84.02 (0.00)	75.55 (0.00)	99.46 (0.00)	15.95 (12.90)	24.42 (21.37)	0.50 (0.22)	0.03	0.03	0.03
vdj^a^	80.51 (0.00)	75.03 (0.00)	96.48 (0.00)	19.49 (4.87)	24.97 (22.51)	3.52 (2.14)	0.00	0.00	0.00
vdjalign^a^	89.21 (0.00)	76.24 (0.00)	99.15 (0.00)	10.79 (4.87)	23.76 (21.41)	0.85 (0.49)	0.00	0.00	0.00
mutated sequences (40)									
IgSCUEAL	99.57 (12.48)	46.95 (6.83)	98.73 (15.50)	0.43 (0.18)	53.05 (50.72)	1.27 (0.77)	0.00	0.00	0.00
IgBLAST	96.05 (9.27)	55.64 (26.32)	94.47 (8.36)	3.95 (0.69)	41.96 (41.50)	5.53 (3.09)	0.00	2.40	0.00
iHMMune	90.90 (0.00)	57.70 (0.00)	92.51 (0.00)	8.47 (0.71)	19.04 (16.26)	6.86 (2.28)	0.63	23.26	0.63
SoDA	91.33 (0.00)	54.95 (0.00)	82.82 (0.00)	8.67 (1.18)	45.05 (41.67)	17.18 (6.00)	0.00	0.00	0.00
V-Quest^a^	96.30 (13.14)	53.87 (0.00)	93.38 (7.16)	3.70 (0.69)	44.52 (40.26)	6.61 (3.85)	0.00	1.61	0.01
Clonanalyst^a^	77.13 (0.00)	58.34 (0.00)	89.20 (0.00)	22.49 (12.77)	41.28 (37.39)	10.43 (1.70)	0.38	0.38	0.38
vdj^a^	75.96 (0.00)	57.35 (0.00)	89.39 (0.00)	24.04 (5.29)	42.65 (39.41)	10.61 (4.20)	0.00	0.00	0.00
vdjalign^a^	83.01 (0.00)	61.48 (0.00)	92.64 (0.00)	16.99 (5.30)	38.52 (35.38)	7.36 (1.87)	0.00	0.00	0.00
mutated sequences (80)									
IgSCUEAL	98.91 (20.80)	26.63 (5.68)	96.62 (25.15)	1.09 (0.61)	73.17 (70.84)	3.18 (2.35)	0.00	0.00	0.00
IgBLAST	91.35 (14.52)	28.84 (18.28)	78.64 (16.99)	8.65 (2.48)	69.64 (69.25)	20.30 (16.40)	0.00	1.52	1.06
iHMMune	74.89 (0.00)	44.91 (0.00)	84.12 (0.00)	20.86 (6.37)	20.21 (17.94)	11.63 (5.84)	4.25	34.88	4.25
SoDA	80.49 (0.00)	26.38 (0.00)	64.13 (0.00)	19.51 (3.36)	73.62 (70.46)	35.87 (22.11)	0.00	0.00	0.00
V-Quest^a^	91.59 (19.41)	33.60 (0.00)	85.70 (10.12)	8.41 (2.37)	63.48 (59.03)	13.96 (8.40)	0.00	2.92	0.34
Clonanalyst^a^	64.24 (0.00)	31.10 (0.00)	72.16 (0.00)	27.59 (12.66)	60.72 (56.93)	19.67 (6.83)	8.17	8.17	8.17
vdj^a^	70.53 (0.00)	31.73 (0.00)	78.02 (0.00)	29.47 (6.67)	68.27 (65.10)	21.98 (11.32)	0.00	0.00	0.00
vdjalign^a^	76.73 (0.00)	38.94 (0.00)	83.30 (0.00)	23.27 (6.49)	61.06 (57.83)	16.70 (6.84)	0.00	0.00	0.00

^a^Limited to the sequences composed of the subset of genes included in the reference set for the method.

Finally, mutations (40 or 80 per sequence, representing roughly 10% and 20% nucleotide divergence from baseline, respectively) were introduced into the rearranged sequences with insertions, deletions and N-nucleotides, using the S5F model proposed by Yaari *et al.* [31]. This is a model that considers the importance of sequence context (two bases either side of a position of interest) in both the rate and the type of mutation. While the simulations consider context-dependence, the substitution model used by IgSCUEAL does not; nevertheless, for mutated sequences, IgSCUEAL outperforms IgBLAST in assigning V and J regions. While performance generally drops with increasing divergence from germline, this effect is not as extreme with IgSCUEAL, such that its advantage over other methods grows with more mutations (table 1). IgSCUEAL maintains excellent performance in the V and J regions, returning the correct allele in the set of credible assignments in 99.7% and 98.8%, respectively, for the 40 mutation dataset, and 98.9% and 98.8% for the 80 mutation dataset. In most cases (90% for V and 83% for J), the correct allele is also the most probable one. When an error is made in V allele classification, in four of five cases the algorithm still finds the correct gene; for J alleles this proportion is about one in three. Sequences simulated with insertions and mutations make homology-based D-region recovery difficult, with only 48.2% of credible assignment sets including the allele used for simulation. The performance of IgSCUEAL matches or is superior to IgBLAST in this setting.

The performance of other methods is shown in table 1. As V-Quest uses the homology searches based on BLAST, its performance tracks that of IgBLAST, albeit a direct comparison is not easily possible, because the implementation of V-Quest does not allow custom reference sets. Somewhat surprisingly, hidden Markov model (HMM) tools (iHMMune, SoDA) achieve uniformly inferior performance in classifying V and J alleles on mutated sequences when compared with homology-based tools, while D region classification results are better for HMM tools in these situations.

We initially hypothesized that higher levels of mutation may result in query sequences clustering with ancestral sequences; however, this was not the case for our simulated data where higher levels of mutation result in larger credible sets comprising alleles from the reference alignments. For the simulations with insertions and deletions, the credible set (i.e. those assignments with Akaike weights ≥0.01) of V regions comprised four alleles (interquartile range, IQR, 3–6), increasing to 6 (IQR 4–9) alleles for the 40 mutation simulations and to 8 (IQR 5–12) alleles for the 80 mutation simulations.

### (b) Sequences from genotyped individuals

The ‘Stanford S22’ dataset comprises 13 153 sequences from an individual who was fully genotyped. The performance of an algorithm is assessed by the proportion of sequences that are assigned to a germline gene that is absent from the individual [32]. IgSCUEAL achieves the lowest rates of V and J misclassification among all methods compared, which are the targets of phylogenetic screening, but performs relatively poorly on D classification, highlighting the shortcomings of naive alignment-based assignment of short D alleles (table 2).

**Table 2.** Comparative model performance for the Stanford S22 dataset [32], based on the number of reads assigned to a germline allele (higher is better), and the percentage errors at either the gene level or the allele level (lower is better).

	reads assigned an allele, %			incorrect gene, %			incorrect allele, %			
method	V	D	J	V	D	J	V	D	J	error, %
IgSCUEAL	100	99.3	100	0.08	2.44	0	2.81	2.59	0.47	8.21
IgBLAST	97.7	97.3	97.3	0.35	2.16	0	3.11	1.80	0.86	8.06
iHMMune	93.5	92.0	93.5	0.21	0.94	0	3.35	1.27	1.95	7.50
IMGT/V-QUEST	100	99.7	99.9	0.25	2.37	0	5.82	2.4	1.57	11.69
SoDA	93.2	93.2	93.2	0.29	6.57	0	2.77	1.62	1.74	12.24
Cloanalyst	99.9	99.9	99.9	0.51	3.37	0	6.82	1.63	1.18	12.82
vdjalign	100	100	100	0.34	1.82	0	9.01	2.33	0.92	13.63

We developed an interactive viewer of assignments and statistics such as the distribution of CDR3 length aggregated across sequences; this can be applied to data on immunoglobulin repertoires, as well as on sequences obtained from different individuals, to understand the composition at the population level. To illustrate the latter (figure 2), we analysed a dataset previously studied by Ohm-Laursen *et al.* [13], comprising 6329 immunoglobulin sequences which, although clonally unrelated, were obtained from individuals who had been selected on the basis of being homozygous for IGHV3-23*01 and were sequenced using IGHV3-23 specific primers. In addition, these individuals were homozygous for IGHJ6*02; hence, the proportion of sequences assigned to IGHV3-23*01 and the proportion of IGHJ6 sequences assigned to IGHJ6*02 indicate the accuracy of the algorithm. This is a challenging task, as IGHV3-23*01 and IGHJ6*02 alleles are difficult to classify, as they are similar to other alleles. In the benchmarking assignment of germline genes, IgSCUEAL maps IGHV3-23*01 to the correct allele with only *ca* 50% support, allocating *ca* 25% support each to IGHV3-23*02 and to IGHV3-23*05. Similarly, after excluding a deletion at the 3′ end, the human IGHJ6*02 allele only differs by a single nucleotide from IGHJ6*01. IgSCUEAL inferred IGHV23*01/J6*02 rearrangement as the most probable one for 49.9% of all the sequences in the file, assigned 99.82% of reads to the IGHV3-23 gene, 77.9% of all reads to IGHV3-23*01, and 99.7% of sequences that mapped to IGHJ6 were assigned to the *02 allele.

### (c) Clonally related datasets

**Figure 3.**
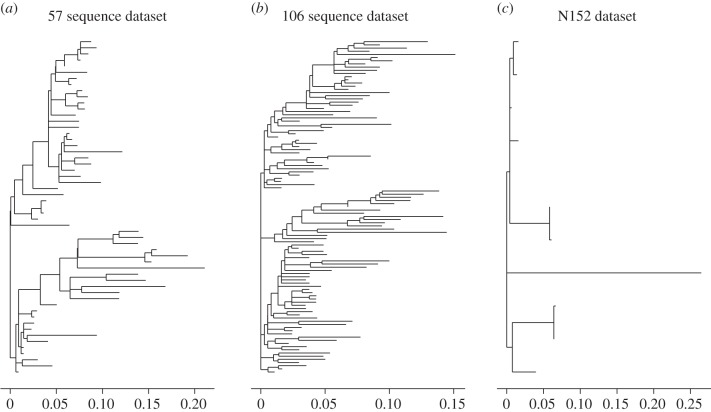
Phylogenetic trees of clonally related IGH sequences, reconstructed by maximum likelihood, and rooted on the centre of the tree (see Material and methods §2g(iii)). These illustrate the high level of genetic diversity in these datasets (13.4%, 11.7% and 12.0% for (*a*), (*b*) and (*c*), respectively), as well as the variable divergence from the root sequence, despite all sequences in a dataset being sampled at the same time. These trees do not illustrate the level of divergence of these sequences from their germline genes (figure 4).

We analysed clonal datasets in order to assess the consistency of assignment for a given clone. Ideally, the different sequences within a clone should share the same V(D)J assignment. As IgSCUEAL generates Akaike weights for a given assignment, assignments for a clone can be combined over the sequences. In addition, we clustered sequences together on the basis of shared V and J alleles in their credible set. Although the true rearrangement is not known with certainty, we also generated a predicted rearrangement for an ancestral reconstruction of the sequences (rooted at the centre of the tree). Figure 3 illustrates that these clonal datasets exhibit significant diversity (*ca* 12% mean pairwise distance, as calculated from the branch lengths of the phylogeny). Hence, using an ancestral reconstruction may help to reduce noise by removing at least some of the somatic hypermutations.

We first analysed a dataset comprising 57 sequences, which was derived from tonsillar B cells that are clonally related. For the ancestral reconstruction, the predicted V/J rearrangement was V3-34*04/J3*02 (52.5% support); there were 10 other rearrangements with support greater than 1%, although all had less than 10% support overall. Individual assignments for each of the 57 sequences were broadly consistent with the assignment of the ancestral sequence; V3-34*04/J3*02 was the commonest ‘best’ assignment (22/57), and also received the highest model-averaged support (26.2%). Although not always the best assignment, by clustering sequences together on the basis of shared V/J assignments, IgSCUEAL clustered 56/57 sequences together (one was too short to return any J assignment), while IgBLAST clustered fewer (54/57) sequences together.

For the 106-sequence dataset, also derived from tonsillar B cells, the difference in support for the ancestral reconstruction between the best rearrangement and the others was lower; the predicted rearrangement was V4-34*01/J6*02 (19.2% support) with 11 other rearrangements with model-averaged support of 1% or more, with the second most supported rearrangement having a support of 10%. V4-34*01/J6*02 was also the best assignment for 63/106 sequences, and had the highest model-averaged support (16.8%). By clustering sequences together on the basis of shared V/J assignments, IgSCUEAL clustered all 106 sequences together, while IgBLAST clustered 104/106.

We also analysed 11 clonal sequences from donor N152, an HIV-infected individual who was the source of the broadly neutralizing antibody 10E8. This antibody is heavily mutated—approximately 20% divergent from germline—to the extent that SoDA was unable to generate assignments for 5/11 sequences. Using IMGT, Huang *et al.* [36] originally identified the heavy chain of 10 ×10^8^ as a V3-15*05/D3-3*01/J1*01 reassortment; for the ancestral reconstruction, IgSCUEAL also supported a V3-15*05/J1*01 reassortment (65% support), although there were five other supported assignments involving other V3-15 alleles, with V3-15*01 having the second highest support (13%). V3-15*05/J1*01 was also the commonest best assignment (5/11), although averaged over the sequences, V3-15*07/J1*01 had the highest model-averaged support (41.5% versus 32.6%). Both V3-15*05 and V3-15*07 were in the set of rearrangements for all sequences, and all rearrangements included J1*01, and hence all sequences clustered together on the basis of shared V/J rearrangements.

We also considered simulated clonal sequences, generated by randomly sampling a ‘seed’ sequence from unmutated rearrangements, and generating multiple sequences with different sets of mutations. For 100 clones, each comprising 100 sequences with 40 mutations (*ca* 10% divergence) from germline, IgSCUEAL clustered all 100 sequences together on the basis of shared V/J assignments for 96 clones, with a cluster size of at least 96 for the four clones where sequences did not all cluster together. This result was robust to the number of mutations (80 versus 40).

**Figure 4.**
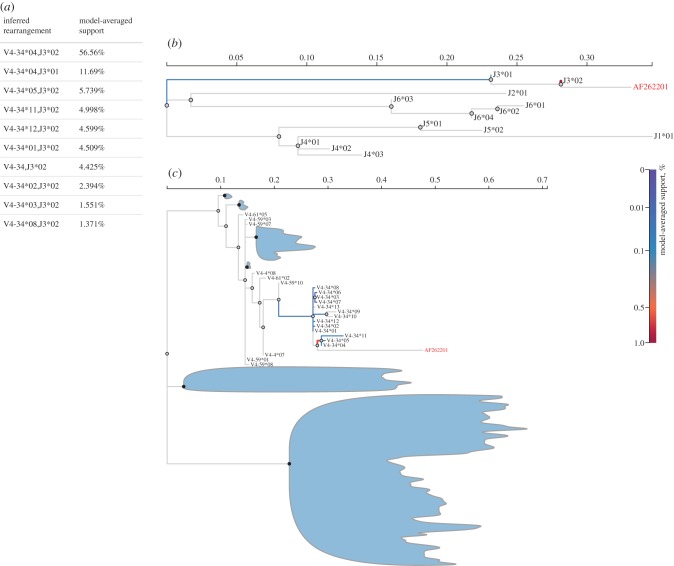
IgSCUEAL classification results for AF262201 from the PW57 dataset. Based on ≈20 000 phylogenetic attachment models examined by IgSCUEAL, the best-supported rearrangement is V4-34*04 (D3-10*01) J3*02. Alternative rearrangements, all involving V4-34 and J3 alleles are shown in panel (*a*). The inferred model-averaged support for attaching the J and V regions of AF262201 to various branches in the reference trees (most of the V tree has been collapsed for clarity) are shown as branch colours in panels (*b*) and (*c*), respectively.

The above clonal datasets illustrate the variable level of ambiguity when assigning reassorted immunoglobulin genes to germline genes, as this is a function both of the level of divergence of the reassorted gene, as well as on the underlying phylogenetic structure of the reference germline sequences. We used the 57 sequence clonal dataset to illustrate how detailed information on the assignment of an individual sequence can be visualized (figure 4). In addition to the inferred phylogenetic placement for V and J regions, the interactive visualization (see the electronic supplementary material) also presents a summary of the inferred rearrangements and their support, as well as amino acid alignments of the FR and CDR in the V region, and the J region of the query sequence and a set of inferred germline alleles.

## 4. Discussion

Through the use of multiple simulated datasets and several biological datasets, we have demonstrated that inclusion of phylogenetic information can result in more accurate assignment of reassorted immunoglobulin heavy (IGH) chains to germline V and J alleles, particularly for mutated rearrangements. The impact of mutations is mostly to increase the uncertainty in assignment, which manifests as either comparable support across multiple alleles, or support for clustering with an ancestral sequence. Further improvements to IgSCUEAL may be possible through the use of more realistic evolutionary models of somatic mutations, e.g. those that consider context-dependent substitution. Like other phylogenetic placement approaches [40], IgSCUEAL comes at an increased computational cost compared with highly tuned similarity-based approaches such as IgBLAST; indeed, of all the approaches, both similarity- and model-based, IgSCUEAL is the most computationally intensive, despite various optimizations in our implementation. Nevertheless, we have employed IgSCUEAL in our own analyses on datasets comprising hundreds of thousands of sequences. If computational resources are limiting, IgSCUEAL may be better placed for confirmatory rather than exploratory analyses. Given that IgBLAST works well for unmutated sequences and is much faster than IgSCUEAL, screening sequences using IgBLAST and determining the extent of mutations may be a useful preliminary analysis prior to using IgSCUEAL.

Although we consider the phylogeny of V and J germline genes, we treat each query sequence independently; this is not the case when studying immunoglobulin repertoires, where there is shared ancestry within a clonotype. Phylogenetic approaches can be used to confirm clonality, through the comparison of the phylogenies for the V, D and J regions, as well as to obtain ancestral reconstructions of the rearranged immunoglobulin, which may help to reduce noise in the V(D)J assignments resulting from somatic hypermutation. The latter approach may be particularly useful if there are repeated samples of a repertoire from multiple timepoints; it may be possible to trace the ancestry of a highly mutated sequence, which is difficult to assign to germline genes, back to a less mutated sequence.

The focus of our approach has been to characterize the V and J regions of reassorted immunoglobulin genes, as these regions can be aligned with a reasonable amount of confidence at the germline level, and are sufficiently long to be able to generate a reference-based phylogeny. For convenience, we provide a simple alignment-based approach to identify the closest matching D allele, but like many other approaches, the accuracy of D region assignment is poor. D regions are short and highly variable at the germline level, and may be obscured by deletions, somatic hypermutation and convergent evolution [41] at the level of the reassorted immunoglobulin gene, such that all traces of the original germline may be obliterated. While it may be infeasible to assume an underlying phylogeny for D germline alleles, a probabilistic model of D region evolution—taking into account, for example, the mutational load in the V and J regions—may help to estimate a credible set of D germline alleles. Other approaches which may result in further improvement to D gene assignment include refinement of scoring schemes based on evaluation datasets, an approach taken by Ab-Origin [8] to improve assignments based on BLAST, or pre-processing of the rearranged immunoglobulin to identify regions using models such as conditional random fields, an approach used by Malhotra *et al.* [42], prior to using a different approach to assign regions to germline alleles.

As a query immunoglobulin sequence can cluster with an ancestral sequence, IgSCUEAL may also be more robust to missing alleles. However, if a gene is represented by only a single allele in the reference set, and there are undescribed alleles, then IgSCUEAL may assign a high support to an incorrect allele, as there will be no ancestral sequence at the allele level. The Akaike weights provided by IgSCUEAL as a measure of support for a given rearrangement are conditional on the reference alignment, and should be interpreted cautiously for species where the immunoglobulin gene diversity has not been fully explored. As the run time of IgSCUEAL is largely independent of the size of the reference dataset used, inclusion of putative germline immunoglobulin sequences, for example V gene like sequences identified in the genomes of mammals [43], can easily be included without resulting in greatly increased computational cost.

With few exceptions (e.g. [28,35]), most studies of immunoglobulin genes have not attempted to use phylogenetic approaches; in part, this stems from misconceptions regarding phylogenetic analysis and model fitting. For example, Chen *et al.* [24] stated:

Standard phylogeny inference methods are not suitable for exploring clonal relationships within an immunoglobulin gene sequence dataset as antibodies diversify through processes that differ substantially from those of long time scale evolutionary events.

However, phylogenetic approaches have been gainfully employed in the study of the evolution of HIV (and other RNA viruses) within an infected individual [44], which is characterized by high rates of mutation, recombination, and selection—hardly less complex than that of immunoglobulin genes. There is also the misconception that phylogenetic approaches do not permit sampled sequences to be the direct ancestors of others [45], or that they cannot be applied when no mutations occur along an internal branch of the tree. Under the maximum likelihood paradigm, these scenarios can be accommodated by incorporating a branch of zero length; this is the approach we take when comparing reassorted immunoglobulin genes to the reference germline genes. Under the Bayesian paradigm, priors have been proposed that allow polytomies [46] and sampled ancestors [47]. While methods have to be adapted to take into account the specifics of the underlying mechanisms, such as evolution of a clone from a state largely determined by the germline, we anticipate further growth of the application of phylogenetic methods in the dynamics of immunoglobulin repertoires.

## Supplementary Material

IgSCUEAL

## Supplementary Material

Evaluation datasets
