# Supplementary material for: Assigning and visualizing germline genes in antibody repertoires
Source: Philos Trans R Soc Lond B Biol Sci. 2015 Sep 5;370(1676):20140240. doi: 10.1098/rstb.2014.0240 (PMC4528417; doi:10.1098/rstb.2014.0240)
Supplement: IgSCUEAL [file rstb20140240supp1.zip › IgSCUEAL-master/Simon/evaluation_sim0.html]

Evaluation - sim0


# Evaluation - sim0

#### *Simon Frost*

#### *12/01/2015*

```
igscueal.name <- "sim0_igscueal_full.txt"
igblast.name <- "sim0_igblast.txt"
```

```
##            Gene IgSCUEAL IgBLAST Total
## 1      V allele     2831    6762  7038
## 2 D gene/allele     5848    6426  7038
## 3      J allele     3222    7038  7038
```
