# Supplementary material for: Assigning and visualizing germline genes in antibody repertoires
Source: Philos Trans R Soc Lond B Biol Sci. 2015 Sep 5;370(1676):20140240. doi: 10.1098/rstb.2014.0240 (PMC4528417; doi:10.1098/rstb.2014.0240)
Supplement: IgSCUEAL [file rstb20140240supp1.zip › IgSCUEAL-master/Simon/evaluation_sim1.html]

Evaluation - sim1 unmutated


# Evaluation - sim1 unmutated

#### *Simon Frost*

#### *12/01/2015*

```
igscueal.name <- "sim1_igscueal_full.txt"
igblast.name <- "sim1_v2_igblast.txt"
```

```
##            Gene IgSCUEAL IgBLAST Total
## 1      V allele      448     939  1000
## 2 D gene/allele      540     699  1000
## 3      J allele      627     994  1000
```
