# Supplementary material for: Assigning and visualizing germline genes in antibody repertoires
Source: Philos Trans R Soc Lond B Biol Sci. 2015 Sep 5;370(1676):20140240. doi: 10.1098/rstb.2014.0240 (PMC4528417; doi:10.1098/rstb.2014.0240)
Supplement: IgSCUEAL [file rstb20140240supp1.zip › IgSCUEAL-master/viz/apps/multiple-tree.html]

 IgSCUEAL analysis result


Toggle navigation

IgSCUEAL tree support

- Summary
- Tree
- Sequences

- Load file

V

J

Toggle unsupported branches
