# Supplementary material for: Assigning and visualizing germline genes in antibody repertoires
Source: Philos Trans R Soc Lond B Biol Sci. 2015 Sep 5;370(1676):20140240. doi: 10.1098/rstb.2014.0240 (PMC4528417; doi:10.1098/rstb.2014.0240)
Supplement: IgSCUEAL [file rstb20140240supp1.zip › IgSCUEAL-master/viz/apps/rearrangement-viewer.html]

 Inferred rearrangement viewer 


Toggle navigation

IGH rearrangement explorer

- Load file

Viewing data on  reads
of which  ()
mapped to the reference,  ()
had support of at least  for the best inferred assignment,
and  () had a productive and
complete junction region.

Distribution of assignments

CDR3 lengths

×

×

×
