# Supplementary material for: Assigning and visualizing germline genes in antibody repertoires
Source: Philos Trans R Soc Lond B Biol Sci. 2015 Sep 5;370(1676):20140240. doi: 10.1098/rstb.2014.0240 (PMC4528417; doi:10.1098/rstb.2014.0240)
Supplement: IgSCUEAL [file rstb20140240supp1.zip › IgSCUEAL-master/viz/apps/single-sequence.html]

 IgSCUEAL analysis result


Toggle navigation

IgSCUEAL results

- Summary
- Tree
- Sequences

- Load file

- ### IgSCUEAL result for

  Inferred a  rearrangement
  with  support.

  Based on the  reference alignment
    
  Please cite PMID XXX if you use this result in a publication, presentation, or other scientific work.

- #### Sequence features

  | Region | Length | Amino-acid sequence |
  | --- | --- | --- |

- #### Credible rearrangements

  | Inferred rearrangement | Model averaged support |
  | --- | --- |
  IgSCUEAL considered  rearranement/breakpoint configurations.

V

J

Protein

Nucleotide

FR1

CDR1

FR2

CDR2

FR3

CDR3

J

| Sequence ID |  |
| --- | --- |
